# Trophism and Homeostasis of Liver Sinusoidal Endothelial Graft Cells during Preservation, with and without Hypothermic Oxygenated Perfusion

**DOI:** 10.3390/biology11091329

**Published:** 2022-09-08

**Authors:** Francesco Vasuri, Giuliana Germinario, Carmen Ciavarella, Michele Carroli, Ilenia Motta, Sabrina Valente, Matteo Cescon, Antonia D’Errico, Gianandrea Pasquinelli, Matteo Ravaioli

**Affiliations:** 1 Pathology Unit, IRCCS Azienda Ospedaliero-Universitaria di Bologna, Via Albertoni 15, 40138 Bologna, Italy; 2 Department of General Surgery and Transplantation, IRCCS Azienda Ospedaliero-Universitaria di Bologna, Via Albertoni 15, 40138 Bologna, Italy; 3 Department of Medical and Surgical Sciences (DIMEC), University of Bologna, 40138 Bologna, Italy; 4 Clinical Pathology, Experimental, Diagnostic and Specialty Medicine Department (DIMES), University of Bologna, IRCCS Azienda Ospedaliero-Universitaria di Bologna, Via Albertoni 15, 40138 Bologna, Italy

**Keywords:** CD34, endothelial cells, ERG, hypothermic oxygenated perfusion, liver transplantation, machine perfusion, nestin, VEGFR

## Abstract

**Simple Summary:**

Little is known about the functions and intracellular mechanisms of the endothelial cells in liver grafts. In particular, we still know little about the effect of the most recent machine perfusion techniques currently applied to improve liver transplant outcomes. In this study we analyzed different endothelial markers of both immunohistochemical and gene expression in two different biopsies (for each donor). We observed a severe depression of endothelial trophism in liver grafts, which is restored after reperfusion. This is interesting for further studies on liver grafts, especially considering that the execution of HOPE seems to improve this functional recovery. We propose that our results may help improve the knowledge on graft tissues in order to customize the perfusion techniques prior to transplant and, therefore, improve liver transplant outcomes.

**Abstract:**

The aim of the present study was to evaluate the homeostasis and trophism of liver sinusoidal endothelial cells (LSECs) in vivo in different stages of liver graft donation, in order to understand the effects of graft ischemia and perfusion on LSEC activity in liver grafts. Special attention was paid to grafts that underwent hypothermic oxygenated perfusion (HOPE). Forty-seven donors were prospectively enrolled, and two distinct biopsies were performed in each case: one allocation biopsy (at the stage of organ allocation) and one post-perfusion biopsy, performed after graft implant in the recipients. In all biopsies, immunohistochemistry and RT-PCR analyses were carried out for the endothelial markers CD34, ERG, Nestin, and VEGFR-2. We observed an increase in CD34 immunoreactivity in LSEC during the whole preservation/perfusion period (*p* < 0.001). Nestin and ERG expression was low in allocation biopsies, but increased in post-perfusion biopsies, in both immunohistochemistry and RT-PCR (*p* < 0.001). An inverse correlation was observed between ERG positivity and donor age. Our results indicate that LSEC trophism is severely depressed in liver grafts, but it is restored after reperfusion in standard conditions. The execution of HOPE seems to improve this recovery, confirming the effectiveness of this machine perfusion technique in restoring endothelial functions.

## 1. Introduction

Vascular endothelial cells represent the interface between blood flow and tissue, exerting several functions beyond a mere barrier, including tissue homeostasis, metabolite and nutrient cross-transport, regulation of inflammation and neoangiogenesis, as well as control of the muscular tone of the vessel wall [1]. Liver sinusoidal endothelial cells (LSECs) show an even higher specialization level, being characterized by discontinuous surfaces, diffuse fenestrae for the passage of macromolecules, and the absence of a basal membrane [2]. LSECs actively cross-talk with hepatocytes, stellate cells, and Kupffer cells, contributing to all liver physiological processes: for instance, LSECs inhibit the profibrotic and vasoconstrictive activity of stellate cells [2]. In chronic liver diseases, in response to direct or indirect harmful stimuli, LSECs undergo so-called “endothelization” (or “capillarization”) [3], with loss of fenestrae and acquisition of a phenotype commonly seen in usual capillaries, among which the expression of CD34 is the most evident, as well as the most useful in histopathological practice [4]. During chronic damage and endothelization, LSEC lose the inhibitory function on stellate cells, moving towards profibrotic and vasoconstrictive activity [2].

In the few last decades, the poor availability of grafts for liver transplantation has led to the search for new strategies to increase the donor pool: one of these strategies is the use of organs from extended criteria donors (ECD). The ECD inclusion criteria are age >60–65 years, and/or the presence of other criteria, such as donors in cardiovascular death (DCD), high transaminase levels or hypernatremia, long intensive care, or prolonged cold ischemia time [5]. Organ preservation is crucial when ECD transplant grafts are utilized, which appear more vulnerable when treated with standard techniques, such as static cold storage (SCS) [6], leading to a high risk of acute and chronic liver injury caused by ischemia–reperfusion after liver transplantation of ECD [7]. The machine perfusion (MP) is a recent organ preservation strategy to increase the survival of organs from ECD: at present, dynamic MP can be performed in hypothermic, subnormothermic, and normothermic conditions [8]. The hypothermic oxygenated perfusion (HOPE) allows the redirection from anaerobic metabolism to aerobic metabolism under hypothermic conditions, and protects grafts from species-related oxidative damage. The superiority of HOPE preservation to simple cold storage was reported in clinical liver preservation studies [9,10,11,12]. LSECs play a key role in the regulation of the venous pressure gradient (at 4 mmHg in normal conditions), by producing vasodilating (nitric oxide synthase, NOS) and vasoconstricting (endothelin-1) factors in response to intrahepatic shear stress [13]: this is of utmost importance, since one of the protective functions of machine perfusion techniques is the maintenance of adequate shear stress, in order to avoid endothelial damages and the shift in balance toward thrombosis and vasoconstriction [14]. The preservation of LSECs during machine perfusion is strongly time-dependent, since it has been demonstrated that LSECs can bear 8–16 h of cold storage, versus the 72 h reported for hepatocytes in vitro, as well as the fact that ischemic damage to LSECs drastically impacts on hepatocyte functional response in vivo [15,16].

The ETS-related gene (ERG) is a transcription factor with an emerging role in the regulation of endothelial functions, during embryogenesis, but also in several adult tissues [17]. ERG expression is constitutively regulated by several genes involved in neoangiogenesis and response to hypoxia, such as NOS, VE-cadherin, and von Willebrand factor, and it is inhibited by proinflammatory stimuli such as TNF-α [18,19,20,21]. Nestin represents another interesting marker of endothelial trophism and intracellular homeostasis regulation: it is a type IV intermediate filament, originally described in nervous stem cells, where it regulates the radial axon growth [22]. Our group studied Nestin expression and localization in the liver, finding a basal immunohistochemical expression in the portal arterioles: sinusoids usually do not show Nestin positivity, with the noteworthy exception of neoplastic sinusoids during hepatocellular carcinoma progression [23,24].

The aim of the present study is to evaluate the expression of different markers of LSECs trophism and homeostasis in vivo at different stages of liver graft donation, before and after HOPE, in order to understand the effects of graft ischemia and perfusion on LSECs.

## 2. Materials and Methods

### 2.1. Donors’ Enrolment

The present study was approved in advance by the local Ethical Committee (code number 65/2018/SPER/AOUBo), and follows the ethical guidelines of the 1975 Declaration of Helsinki (6th revision, 2008); donors are kept anonymous. The study includes donors previously published in the context of a larger open-label, prospective, single-center, randomized clinical trial, where patients were stratified based on the contemporary presence of ECD liver criteria and randomized in a 1:1 ratio to receive a liver preserved with either HOPE after SCS during transportation or with SCS alone [9,25]. Livers undergoing SCS were stored in sterile organ bags with Belzer or Celsior solution, and cooled on ice as previously described and according to the Center policy [9,10,25]. HOPE was performed through the portal vein at a pressure of 5 mmHg by flushing the organ at low flow values (30 mL/min) with new oxygenated perfusion fluid (Belzer MPS) during back-table preparation with the aim of removing waste products and residual microthrombi, and to provide oxygen. Successively, the organs were treated with continuous HOPE until the grafts were transplanted.

An eligibility criterion specific for the present study was the availability of two distinct biopsies:

1. Allocation biopsy (A-Biopsy), performed at the stage of organ allocation for the assessment of graft suitability, as usually performed in our Institution [26];
2. Post-perfusion biopsy (PP-Biopsy), performed for the purposes of the study after graft implant in the recipients. This biopsy was specifically performed for the study.

Of the 110 liver donors prospectively enrolled in the context of the monocentric clinical trial, 49 satisfied the eligibility criteria; two more donors were excluded due to the lack of sufficient tissue in A-Biopsies to perform IHC: 47 donors were therefore finally enrolled for the current study, 22 males and 25 females, with a mean age of 74.8 ± 10.1 years (range 42–87 years).

Of these 47 donors, 34 (72.3%) donors were enrolled for HOPE perfusion, while 13 (27.7%) were preserved in SCS.

Collected donor variables included tobacco and/or alcohol consumption, chronic use of medications; heart, lung, and liver disease; hypertension; diabetes; dyslipidemia; nephropathy; vasculopathies; dyslipidemia; and body mass index (BMI). Machine perfusion variables included flow, pressure, and resistance; gas analysis variables of the effluent perfusate included PH, pCO2, paO2, glucose, and lactate at start of perfusion, and then every 30 min.

Analysis of recipients’ characteristics was not included in the present study due to the study aims and the small sample size; however, a follow-up of the recipients was preliminarily included after a mean follow-up of 305.6 ± 193.5 days (range 45–723 days). Follow-ups included early allograft dysfunction (EAD) and primary non-function (PNF) cases, post-transplant acute kidney injury (AKI) with or without the need of continuous venovenous hemofiltration (CVVH), graft survival, and recipient survival.

### 2.2. Histopathological Analysis and Immunohistochemistry

Different endothelial markers were applied to evaluate the trophism of LSECs before and after perfusion. The term “trophism” was used to indicate the activation of those intracellular pathways leading to cell protection and/or survival in course of hypoxic stresses.

A-Biopsies were routinely frozen for quick histopathological evaluation for graft suitability, and subsequently fixed in formalin and embedded in paraffin (FFPE); PP-Biopsies were directly FFPE at the stage of sampling. In all cases, FFPE tissue was routinely processed, and 2-µm-thick sections were cut for hematoxylin–eosin and reticulin silver staining, as well as for immunohistochemistry (IHC). The histopathological variables collected are the same of the donor’s checklist usually applied in our institution [26]: portal fibrosis stage according to Ishak [27], lobular fibrosis (absent, focal, diffuse), portal and lobular inflammation according to Ishak, lobular necrosis according to Ishak, myointimal thickening of hepatic arteries and arterioles (absent, mild, moderate, and severe), bile duct regression (absent, present) with or without bile duct reaction, percentage of macrovesicular and microvesicular steatosis, cholestasis (absent, mild, moderate, severe).

IHC was automatically performed by means of the automated immunostainer Benchmark^®^ ultra (Ventana Medical Systems, Inc, Roche group, Tucson, AZ, USA), following the manufacturer’s instructions. The antibodies used in the present study included: CD34 (clone QBEnd/10), Nestin (clone 10C2), and ERG (clone EPR3864). CD34 immunoreactivity was semiquantitively assessed in LSECs as mild/focal, moderate, and severe/diffuse, based on the extension of sinusoids “endothelization” from the periportal zone to the whole lobule. Nestin immunoreactivity was evaluated at two levels: (1) semiquantitively at LSEC level (when Nestin-positive sinusoids were present), applying the same method as CD34; (2) quantitatively counting the highest number of periportal Nestin-positive capillaries, which we observed to be common in our series. ERG immunoreactivity was quantitatively assessed by counting the number of ERG-positive endothelial nuclei in 10 high-power fields (HPF, 40× magnification): the mean number of ERG-positive nuclei/10 HPF was therefore counted. As controls, two liver graft specimens from healthy living donors (age 31 and 39 years) were used: in these cases, no CD34-positive endothelization of LSEC and no significant Nestin immunoreactivity were observed (as expected); the mean number of ERG-positive nuclei was 13.3 and 14.5/10 HPF respectively.

### 2.3. RNA Extraction and Reverse Transcriptase-Polymerase Chain Reaction (RT-PCR)

Total RNA was extracted from 16 FFPE tissue samples, eight HOPE and eight SCS cases, from both A-Biopsies and PP-Biopsies, using the FFPE Recover All (Thermo Fisher Scientific, Waltham, MA, USA) according to the manufacturer’s instructions. RNA quality and concentration was evaluated by a ND-1000 spectrophotometer (NanoDrop, Thermo Fisher Scientific, Waltham, MA, USA). Reverse transcription was performed from 0.5 μg of total RNA in 20 μL reaction volume using the High-Capacity cDNA Reverse Transcription Kit (Thermo Fisher Scientific). Real-Time PCR for the analysis of Nestin, ETS Transcription Factor (ERG) and Vascular Endothelial Growth Factor Receptor-2 (VEGFR-2) was assessed in a CFX Connect Real-Time PCR Detection System (Bio-Rad Laboratories. Hercules, CA, USA) using the SYBR green master mix (Bio-Rad Laboratories). Primer sequences were designed through the NCBI primer tool and were the following: Nestin FWD GACCCTGAAGGGCAATCACA; Nestin REV GGCCACATCATCTTCCACCA; ERG FWD TCGCATTATGGCCAGCACTA, ERG REV CGTTCCGTAGGCACACTCAA; VEGFR-2 FWD CGGTCAACAAAGTCGGGAGA; VEGFR-2 REV CAGTGCACCACAAAGACACG (Sigma-Aldrich. St. Louis, MO, USA). Reactions were performed in triplicate and target gene expression was normalized to glyceraldehyde 3-phosphate dehydrogenase (GAPDH). Relative quantification was assessed by the comparative 2^−∆∆Ct^ method and data were expressed as fold changes of mRNA expression relative to A-Biopsies.

### 2.4. Statistical Analysis

Variables were reported as means ± standard deviation, ranges, and frequencies. Statistical analysis was carried out using SPSS^®^ software for Windows, ver. 20. When applicable, *t*-tests, ANOVA, and non-parametric Mann–Whitney and Kruskal–Wallis tests were used to compare the variables. *p* values <0.05 was considered statistically significant.

## 3. Results

### 3.1. Donor and Graft Characteristics

Mean donors’ BMI was 26.8 ± 4.5 (range 19.9–46.7), tobacco use was recorded in 17 (36.2%) cases, history of alcohol abuse in one (2.1%) case, cardiopathies were recorded in 18 (38.3%) cases, pneumopathies in 10 (21.3%), chronic hepatopathies in two (4.3%), chronic nephropathies in four (8.5%), hypertension in 30 (63.8%), diabetes in six (12.8%) donors (one of which insulin-dependent), dyslipidemia in 16 (34.0%) cases, and arterial and venous vasculopathies in eight (17.0%) and four (8.5%) donors, respectively; 37 (78.7%) donors chronically used medications (mostly antihypertensives).

Recipient follow-ups recorded seven (14.9%) cases of EAD, no cases (0%) of PNF, and 15 (31.9%) AKI cases, with seven (14.9%) cases needing CVVH. Only two recipients died in the follow-up, and only one needed retransplantation.

The main histopathological characteristics of the 47 liver grafts enrolled are listed in Table 1. Both A-Biopsies and PP-Biopsies were separately revised by two dedicated pathologists, blind to each other: no substantial differences were observed between A-Biopsies and the corresponding PP-Biopsies as far as histopathology is concerned. In case of discordance between the two biopsies concerning single variables, PP-Biopsy characteristics were chosen, due to the better tissue quality and lack of freezing artifacts [28]. In the 34 grafts perfused with HOPE, all the perfusion variables concerning the overall procedure were recorded as well, including both the flushing and the perfusion phases, according to the study protocol (Supplementary Table S1) [9].

### 3.2. Modifications of Endothelial Trophism in Allocation and Post-Reperfusion Biopsies

**Figure 1:**
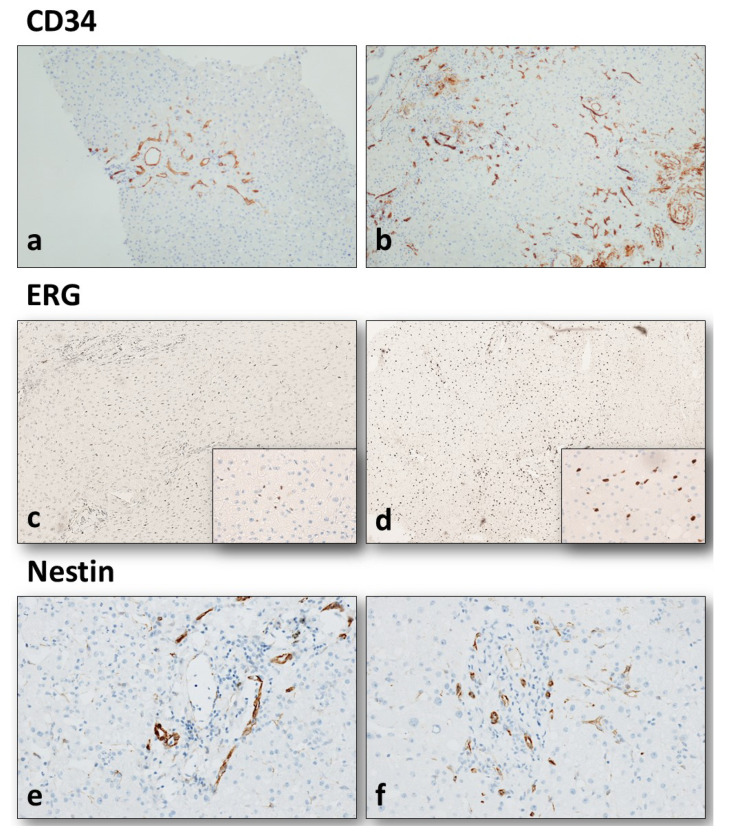
Immunohistochemistry results. CD34 immunoreactivity increased in liver sinusoids from allocation biopsy ((**a**), 2× magnification) to post-perfusion biopsy ((**b**), 2× magnification), as a progressively increasing periportal endothelization (*p* < 0.001). The increased expression of ERG was also statistically significant (*p* < 0.001) between allocation biopsy ((**c**), 2× magnification) and post-perfusion biopsy ((**d**), 2× magnification). The higher density of ERG-positive nuclei is highlighted by higher magnification (small squares, 20× magnification). Two portal tracts immunostained by Nestin ((**e**,**f**), 10× magnification), showing a variable number of periportal capillaries and a mild immunoreactivity in adjacent sinusoid.

In A-Biopsies, sinusoidal CD34 expression was mild/focal in 25 (53.2%) cases, moderate in 13 (27.7%), and diffuse in nine (19.1%). In PP-Biopsies, LSEC CD34 expression was mild/focal in five (10.6%) cases, moderate in 20 (42.6%), and diffuse in 22 (46.8%). We therefore observed an increase in CD34 immunoreactivity in graft sinusoids, mean 0.7 ± 0.9 points, as confirmed by the fact that the “diffuse” cases increased from 19.1% to 46.8%. This difference was statistically significant (*p* < 0.001, *t*-test; Figure 1a,b).

**Figure 2:**
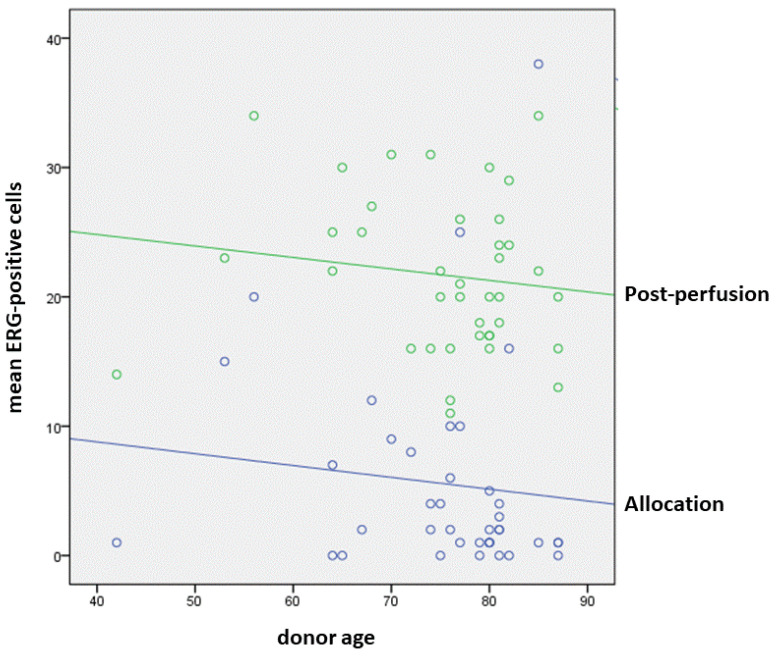
In post-perfusion biopsies (green dots and line), ERG significantly increased compared to allocation biopsies (blue dots and line), but in both groups, an inverse correlation was observed between ERG immunoreactivity and donor age.

The mean number of ERG-positive nuclei in A-Biopsy LSECs was 4.9 ± 7.5, with a maximum number ranging from 1 to 48 (mean maximum number 11.7 ± 12.5). The mean number of ERG-positive nuclei in PP-Biopsy sinusoidal endothelial cells was 22.0 ± 5.8, with a maximum number ranging from 13 to 47 (mean maximum number 28.3 ± 8.4). The increased expression of ERG was statistically significant (*p* < 0.001, Mann–Whitney test); however, an inverse correlation was observed between ERG positivity (on both biopsies) and donor age, as represented in Figure 2 (*p* = 0.031, ANOVA test; Figure 1c,d).

Mild Nestin immunoreactivity was observed in LSECs in 6 (12.8%) A-Biopsies, with a low mean number of Nestin-positive periportal capillaries (mean 1.8 ± 2.6 positive periportal capillaries in hot spots). Nestin immunoreactivity in PP-Biopsy LSECs was absent in six (12.8%) cases, mild in 21 (44.6%), moderate in 14 (29.8%), and diffuse in six (12.8%). The mean number of Nestin-positive periportal capillaries was 7.4 ± 4.2 in hot spots (range 0–24). As for the first two markers, this increased Nestin expression from A-Biopsies to PP-Biopsies was statistically significant (*p* < 0.001, *t*-test; Figure 1e,f).

**Figure 3:**
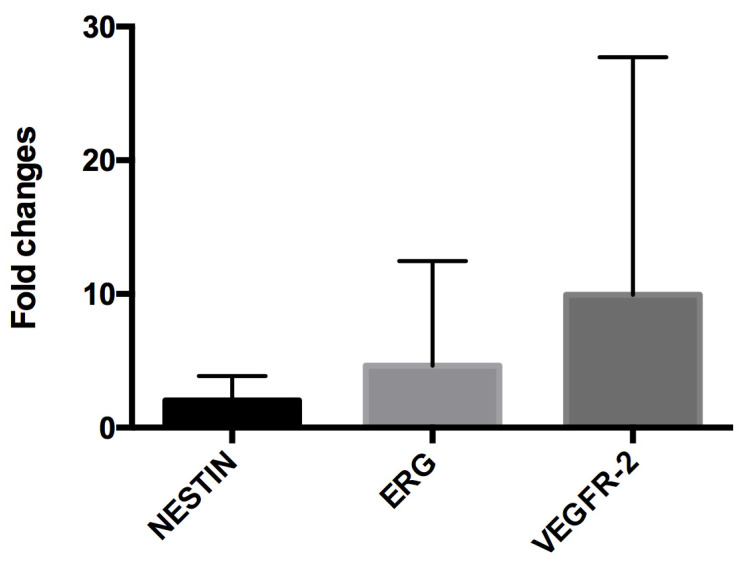
At RT-PCR analysis the mean fold increase from allocation biopsies to post-perfusion biopsies for was 2.04 for Nestin, 4.63 for ERG, and 9.95 for VEGFR-2.

RT-PCR analysis confirmed the induction of the intracellular endothelial pathways suggested by IHC: the mean fold increases between A-Biopsies and PP-Biopsies for VEGFR-2, ERG and Nestin genes were 9.95 ± 17.77, 4.63 ± 7.83, and 2.04 ± 1.83, respectively (Figure 3). Notably, no differences in IHC expression were found between grafts perfused with HOPE and grafts preserved in SCS (see also Table 2), while in RT-PCR, the increase expression of VEGFR-2 was significantly higher after HOPE (mean 15.09) than after SCS (mean 1.36, *p* = 0.025, Kruskal–Wallis test), suggesting a beneficial role of HOPE in terms of endothelial cell homeostasis and survival.

### 3.3. Perfusion Characteristics Influencing Endothelial Trophism in Liver Grafts and Preliminary Follow-Up Results

Among the 34 grafts who underwent to HOPE prior to liver transplantation, some perfusion characteristics correlated with the expression of the three endothelial markers. In particular, the increase in CD34 immunoreactivity in LSECs (endothelization) positively correlated with the time of the overall perfusion (*p* = 0.047, Spearman’s test). The number of Nestin-positive periportal capillaries in PP-biopsies was positively correlated with the Lactate concentration (*p* = 0.008), and negatively correlated with the pH (*p* = 0.005) at the beginning of the re-cycle. Finally, the increase in the number of ERG-positive nuclei in PP-Biopsies positively correlated with the pO2 at the end of re-cycle (*p* = 0.044).

The only follow-up data that showed a correlation with graft endothelial trophism was the need for CVVH after transplant, which directly correlated with CD34 positivity in PP-Biopsies, suggesting a correlation between LSEC endothelization and kidney function in liver transplant recipients. In particular, all seven CVVH cases showed strong and diffuse positivity for CD34 at PP-Biopsy (*p* = 0.017, *t*-test).

## 4. Discussion

The present study aimed to evaluate the trophism in LSECs in vivo during liver graft procurement, preservation, and perfusion with HOPE. For this purpose, we carried out a morphological, IHC, and RT-PCR analyses on different endothelial markers on graft tissue before and after HOPE, as well as before and after SCS. Our results show an increase in LSEC CD34 immunoreactivity, which occurred invariably during the whole preservation/perfusion time: in the grafts perfused with HOPE, this endothelization is proportional to the overall perfusion time, but it does not seem to correlate to any of the other variables. CD34 immunoreactivity is a sign of so-called LSEC “endothelization”, and even in the present context, it should be considered a sign of protection from a mechanic—rather than biologic—stimulus. Nonetheless, capillarized LSECs have been recently shown to produce extracellular matrix, thus demonstrating an indirect link between endothelization and so-called endothelial–mesenchymal transition (EndMT) [29]. EndMT is a model proposed to describe all those processes during which the endothelial cells lose their phenotype and normal functions in response to pathological noxae, to acquire a less differentiated mesenchymal phenotype, characterized by fusiform cell shape and extracellular matrix production, among others [30]. Hypoxia and inflammation have already been reported to stimulate EndMT; thus, this process is likely to play a role in regulating LSEC function during liver graft ischemia and reperfusion [30]. This setting fits well with the more “traditional” view of LSEC endothelization as a sort of metaplastic shift of endothelial cells towards a less specialized phenotype as a protection from high portal pressure, as it is demonstrated by the diffuse LSEC endothelization in course of cirrhosis [3,4]. The finding of a correlation between post-perfusion CD34 immunoreactivity and the need of CVVH in recipients reinforce further the importance of future studies on intrahepatic resistance and pressure of the grafts, both during HOPE and after transplantation.

In this study, we used Nestin as a more specific marker of EndMT: Nestin positivity is not normally observed in LSECs, as already reported by our group [23], as well as demonstrated by our controls. In A-Biopsies, the Nestin positivity generally remained very low, concerning both staining intensity and diffusion. Conversely, an increase in Nestin expression was observed in LSECs from PP-Biopsies with both IHC and gene expression: a periportal Nestin-positive microcirculation became visible in most cases, and RT-PCR demonstrated a twofold increase in Nestin mRNA from A-Biopsies to PP-biopsies. In addition, we observed a higher Nestin expression in HOPE grafts with higher lactate and lower pH at the beginning of the re-cycle. All these observations highlight a possible role of Nestin in EndMT as an adaptative mechanism of liver microcirculation during ischemia time and reperfusion.

ERG is a known mediator of endothelial trophism, essential for physiological and pathological neoangiogenesis [31], and it is generally expressed in normal endothelial cells [32], as also observed in our controls. ERG has been described to prevent the activation of the prothrombotic pathway during low shear stress (as in cold storage) [30], as well as to be involved in the regulation of the genes of the TGF-β family by enhancing Smad1 and reducing Smad2 and 3 activities [33]: through these mechanisms, ERG protect the endothelial cells from EndMT [34]. In addition, ERG inhibits EndMT by regulating several genes such as TGF-β, FLI1, Notch, and others [18,19,20,21,33]. ERG has been demonstrated to induce endothelial differentiation of non-vascular amniotic cell in vitro, actually reversing EndMT process [34]. An interesting finding in our study is the very low number of ERG-positive nuclei in A-Biopsies, which was further confirmed via gene expression with RT-PCR, followed by a total recovery after reimplantation (on PP-Biopsies). Unlike the former two markers, which increased as a response to mechanical and/or metabolic needs during ischemia and perfusion, the recovery of ERG expression is to be considered as restoring normal—and sometimes greater—endothelial trophism. This is also confirmed by our RT-PCR results, which showed a 4.6-fold increase in ERG mRNA in the PP-Biopsy, and a nearly 10-fold increase in VEGFR-2, which is a major vascular growth factor that works together with ERG in all neoangiogenetic processes, via the VEGF/MAPK/ERG pathway [17]. Notably, the VEGFR-2 increase was significantly higher after HOPE than after SCS: in the present study, the sample size and the low number of main events (two recipient deaths and one graft loss) prevents us from conducting a survival analysis (which was out of the study aims), but our results highlight once more the safety and the efficacy of HOPE at least in restoring LSEC functionality and cellular viability. The efficacy of HOPE in terms of short- and medium-term follow-up was reported in previous studies from our group [9,10].

Another interesting finding is that this “recovery” was inversely correlated to donor age, suggesting a physiological unbalance in ERG activities with ageing. This is in line with previous observations on the progressive lack of LSEC fenestrations, in which sinusoidal dysfunction and increased hepatic microvascular resistance correlated with age [35,36]. The comprehension of the effects of age in liver grafts is of utmost importance, since the use of increasingly older ECDs is becoming the rule in transplant centers.

## 5. Conclusions

In conclusion, our results showed that LSEC trophism is severely depressed in liver grafts, but it is restored after reperfusion in standard conditions. The execution of HOPE seems to improve this recovery, confirming the effectiveness of this MP technique in restoring endothelial functions. Whether this LSEC increases functionality impacts on graft survival and transplant outcomes will be the topic of further research.

## Figures and Tables

**Table 1:** Histopathological features of the 47 liver grafts evaluated.

Histopathological Variable	No. (Percentage)
Portal Fibrosis (Ishak’s stage) 0 1 2 3	3 (6.4%) 19 (40.4%) 23 (48.9%) 2 (4.3%)
Lobular Fibrosis Absent Mild/focal Severe/diffuse	31 (66.0%) 12 (25.5%) 4 (8.5%)
Portal Inflammation Absent Mild Moderate-to-severe	7 (14.9%) 35 (75.5%) 5 (10.6%)
Myointimal Thickening Absent/mild Moderate Severe	22 (46.8%) 17 (36.2%) 8 (17.0%)
Biliocyte/bile duct regression Absent Mild Severe	5 (10.6%) 38 (80.9%) 4 (8.5%)
Lobular Inflammation Absent Mild Moderate-to-severe	30 (63.8%) 15 (31.9%) 2 (4.3%)
Lobular NecrosisAbsent Mild Moderate-to-severe	33 (70.2%) 11 (23.4%) 3 (6.4%)
Cholestasis Absent Mild	36 (76.6%) 11 (23.4%)
Microvesicular steatosis (mean)	6.5 ± 6.7% (0–20%)
Macrovesicular steatosis (mean)	4.2 ± 6.1% (0–25%)

**Table 2:** General, immunohistochemical and main follow-up of the patients enrolled in the HOPE and static cold storage (SCS) groups. * Chi-square test.

	HOPE (n = 34)	SCS (n = 13)	Sig.
Mean Age	73.7 years	78.8 years	n.s.
Sex (male)	17 (50%)	5 (38.5%)	n.s.
MELD score	14.4	23.0	n.s
Cold ischemia time (min)	411.2	361.8	n.s.
			
Post-perfusion CD34 (diffuse endothelization)	14 (41.2%)	7 (53.8%)	n.s.
Post-perfusion mean Nestin-positive capillaries	7.3	7.5	n.s.
Post-perfusion mean ERG-positive sinusoids	21.6	23.2	n.s.
			
Early allograft dysfunction	3 (8.8%)	4 (30.1%)	*p* = 0.064 *
Primary non-function	0	0	n.s.
Graft failure (retransplantation)	0	1	n.s.
Recipients’ death	1	1	n.s

## Data Availability

Not applicable.

## Supplementary Materials

The following supporting information can be downloaded at: https://www.mdpi.com/article/10.3390/biology11091329/s1, Table S1: Perfusion variables.
