# Supplementary material for: Trophism and Homeostasis of Liver Sinusoidal Endothelial Graft Cells during Preservation, with and without Hypothermic Oxygenated Perfusion
Source: Biology (Basel). 2022 Sep 8;11(9):1329. doi: 10.3390/biology11091329 (PMC9495341; doi:10.3390/biology11091329)
Supplement: Supplementary file 1 [file biology-11-01329-s001.zip › biology-1849908-supplementary.pdf]

		Flushing				Perfusion			
		Min	Max	Mean	St.Dev	Min	Max	Mean	St.Dev
**pH**	**Initial**	6.60	7.39	7.02	0,13	6.95	7.34	7.17	0.11
	Final	6.90	7.39	7.04	0.10	6.87	7.25	7.11	0.08
**pCO2**	**Initial**	5	9	5	0.98	4	15	6	2.46
	Final	5	15	8	2.18	5	16	7	2.33
**pO2**	**Initial**	205	279	243	16.75	14	1000	322	205.81
	Final	127	695	236	110.05	89	900	403	233.23
**Glucose**	**Initial**	3	157	13	28.23	181	368	204	33.78
	Final	64	348	152	53.2	185	560	270	79.71
**Lactose**	**Initial**	0.2	2.4	0.3	0.39	0.4	6.1	1,7	1.38
	Final	0.3	7.4	2.4	1.21	0.6	9.2	3,5	1.76

Table S1. Perfusion variables.
